# Ultrafine “dry vapor” hypochlorous: proof-of-concept disinfection application

**DOI:** 10.3389/fcimb.2026.1679518

**Published:** 2026-05-28

**Authors:** Reed B. Hogan, Eleni Mijalis, Charlotte Green, Terrell Porter, Christopher Spankovich

**Affiliations:** 1 GI Associates, GI Alliance, Jackson, MS, United States; 2 Department of Otolaryngology Head and Neck Surgery, University of Mississippi Medical Center, Jackson, MS, United States

**Keywords:** disinfection, dry vapor, healthcare-associated infections, hypochlorous acid, terminal clean

## Abstract

**Introduction:**

Current disinfection strategies are limited by cost, safety concerns, and logistical inefficiencies, particularly in busy healthcare environments. Hypochlorous acid (HOCl) offers broad-spectrum antimicrobial activity with a well-established safety profile, but its use as an ultrafine aqueous “dry vapor” has not been widely studied in healthcare settings. This proof-of-concept study evaluated the feasibility of using ultrafine aqueous HOCl for large-scale disinfection in outpatient medical facilities.

**Methods:**

Four outpatient facilities ranging from 4,500 to 90,000 square feet were treated with ultrafine aqueous (<2 micron) HOCl. Samples were collected from staff, procedure, and public areas. ATP bioluminescence in Relative Light Units (RLU) was measured before treatment and at 30 and 60-minutes post-treatment. Results: Statistically significant reductions in RLU were observed at 30 minutes (p < 0.001) and 60 minutes (p < 0.001) post-treatment compared to baseline. Significant reduction was also noted from 30 minutes to 60 minutes (p < 0.001). All facilities achieved disinfection levels below the guidelines for terminal disinfection of hospital non-surgical areas.

**Conclusion:**

Ultrafine aqueous HOCl is an efficient and safe disinfection method for large medical facilities, with implications for improving infection control practices and reducing healthcare-associated infections.

## 1 Introduction

Healthcare-associated infections and surgical site infections result in increased patient morbidity, mortality, and health care expenditures (Shafer et al., 2019). Despite improved practices, antibiotic use restrictions, and proactive interventions in health care facilities, these infections are becoming more frequent (Ejerhed et al., 2020). The rise of disinfectant-resistant organisms with the ability to inhabit surfaces for extended periods of time and recent COVID-19 pandemic understate the need for improvement in current methods of disinfection in the medical arena (Jinadatha et al., 2024).

The effectiveness of existing disinfection methods is often hindered by high costs, time demands, personnel shortages, and variability in the execution of cleaning protocols (Boyce, 2016). Many commercially available solutions require personal protective equipment given their harsh effects on the skin and respiratory tract. Others require vacating personnel and patients, particularly with aerosolized disinfectants like hydrogen peroxide and ozone, or electronic devices such as ultraviolet light (Overholt and Jones, 2021; Gessi et al., 2023).

Hypochlorous acid (HOCl) is a natural antimicrobial agent produced in mammalian white blood cells in response to injury or infectious insult. Early use as a disinfectant was first documented during the 1918 Influenza pandemic, when employed for air purification in cotton mills and during World War I to treat open wounds (Smith et al., 1915; Masterman, 1941). HOCl is effective against a broad spectrum of pathogens, exhibiting fungicidal, bactericidal, and virucidal properties. Its efficacy has been demonstrated against common problematic organisms, including methicillin-resistant Staphylococcus aureus, Pseudomonas, Clostridium difficile (including spores), COVID-19, influenza A, and RSV, and it is believed to be more effective than bleach and chlorine (Smith et al., 1915; Masterman, 1941; Urushidani et al., 2022; Khalaf et al., 2023) Furthermore, HOCl has robust antimicrobial activity against biofilms, including fungi and common molds such as Aspergillus, Candida albicans, and Mucor indicus (Odorcic et al., 2015). Unlike other agents, it does not lead to the formation of “super” bacteria that are resistant to disinfecting practices.

HOCl is widely used in pure form in cosmetics, eye drops, dental care, and the agricultural industry. HOCl is not considered hazardous for environmental disinfectant use. Adverse events are limited to mild skin and eye irritation, though allergic reactions have been reported (Odorcic et al., 2015; Block and Rowan, 2020; World Health Organization, 2021; Urushidani et al., 2022; Khalaf et al., 2023; Annihlare Medical Systems, 2024). During the recent COVID-19 pandemic, aerosolized HOCl sprays were incorporated globally and recognized by the World Health Organization as potent and environmentally safe disinfectant (Benedusi et al., 2022). Nonetheless, a problem with aerosol HOCl application is that the mist, being over 20 microns in size, is heavy and results in a wet application. Wet applications can lead to corrosion and pose safety risks for critical medical electronic devices. The “mist” application requires significant manpower and is labor intensive and costly.

Alternative to wet application, HOCl can be deployed as an ultrafine aqueous droplet (< 2 micron), referred here as a “dry vapor” state, which is far less corrosive than traditional wet applications and is also low-cost and easy to manufacture (Block and Rowan, 2020). Advancements in manufacturing, process control, pH stabilization, and storage methods have significantly extended its shelf life (Overholt and Jones, 2021). The purpose of this study is to examine the feasibility of dry vapor HOCl for widespread, efficient disinfection in both small and large medical facilities.

## 2 Materials and methods

### 2.1 Study sites and sample collection

Four outpatient facilities (urgent care facility, orthopedic, endoscopy, and ophthalmology surgical centers) were evaluated for this proof-of-concept study. Facility sizes ranged from 4,500 to 90,000 square feet. ATP bioluminescence was used to assess the efficacy of disinfection. Samples from staff areas, procedure areas, and public areas were taken from various surfaces, including door handles, desk surfaces, keyboards, kiosks, phones, and medical equipment. Samples were acquired from 10 x 10 cm surface areas as recommended by manufacturer of ATP monitoring system. Consistent swabbing technique were reviewed with study team. Irregular surface areas (e.g. door knob) were swabbed to approximate 10 x 10 cm area. Samples were collected near the end of the workday, while patients and staff were still present and before customary cleaning. To preserve surfaces, sample sites were clearly marked, areas monitored by study personnel, and remaining staff and patients were instructed to avoid areas. Sites were tagged and coded so repeated samples could be accurately obtained and recorded at same surface location. ATP samples were obtained pre-HOCl application, then at 30 minutes and 60 minutes post-delivery of HOCL. A control study with no application of HOCl was limited to Site 1 and completed on a separate study day following the same protocol. Twenty-five samples at baseline, 30 minutes, and 60 minutes confirmed steady ATP levels. Flame spectrometry was used to determine the distribution of HOCl dry vapor during application. The air-exchange rate based on size of buildings informed duration of exposure. The entire 90,000 sq. ft. building (1 million cubic feet; Site 1) was filled with the dry vapor HOCl in 90 minutes, while the smaller facilities (4,500 Site 2, 5,000 Site 3, and 50,000 Site 4 square feet) were covered in 12, 15, and 30 minutes, respectively. HOCl application required one person, one device, and no PPE.

### 2.2 ATP monitoring

The Hygiena ATP Monitoring System (Delaware) was utilized according to manufacturer guidelines (https://www.hygiena.com/instruments-and-automation/monitoring-systems/hygiena-systemsure-plus). The Hygiena ATP Monitoring Systems measure ATP by a reaction with luciferin enzymes that produces light proportional to the contamination level and displayed in Relative Light Units (RLU). Baseline ATP samples were obtained and then re-tested at 30 minutes and 60 minutes after the dry vapor had filled the entire volume of the facility. ATP RLU levels below 50 is considered acceptable in most healthcare facilities as “disinfected” and consistent with terminal cleaning standards. ATP RLU levels of 10 or less align with operating room disinfection standards.

### 2.3 Disinfection

This study used the Nebula One™ Ultra Dry-Mist Nebulizer from Arcus Manufacturing generates and disperses HOCl molecules smaller than 2 micron droplets into the atmosphere using a patented droplet generation process. Annihilyte HOCL solution (EPA registration number 92449-1) was acquired from Annihilare (Linconlnton, NC) for application. The solution is a dilute form of HOCl at 500 parts per million (ppm) that is vaporized to a concentration of < 300 μg/m^3^ (Annihlare Medical Systems, 2024). The nebulization procedure does not require any modifications to the heating, ventilation, and air conditioning equipment at the facilities and does not lead to moisture accumulation on cleaned surfaces.

### 2.4 Statistical analysis

Descriptive analysis was used including measures of mean, variance, and standard error of mean (SEM). The Kolmogorov-Smirnov test of normality showed the data did not fit a normal distribution; therefore, non-parametric tests were applied. Related-samples Wilcoxon signed rank test was used to determine statistical significance of treatment by comparing pre- and post-treatment periods. Independent-Samples Kruskal-Wallis tests were used to compare baseline levels and the amount of shift that was observed at each site. An alpha of <0.05 was used to determine statistical significance.

## 3 Results

**Figure 1:**
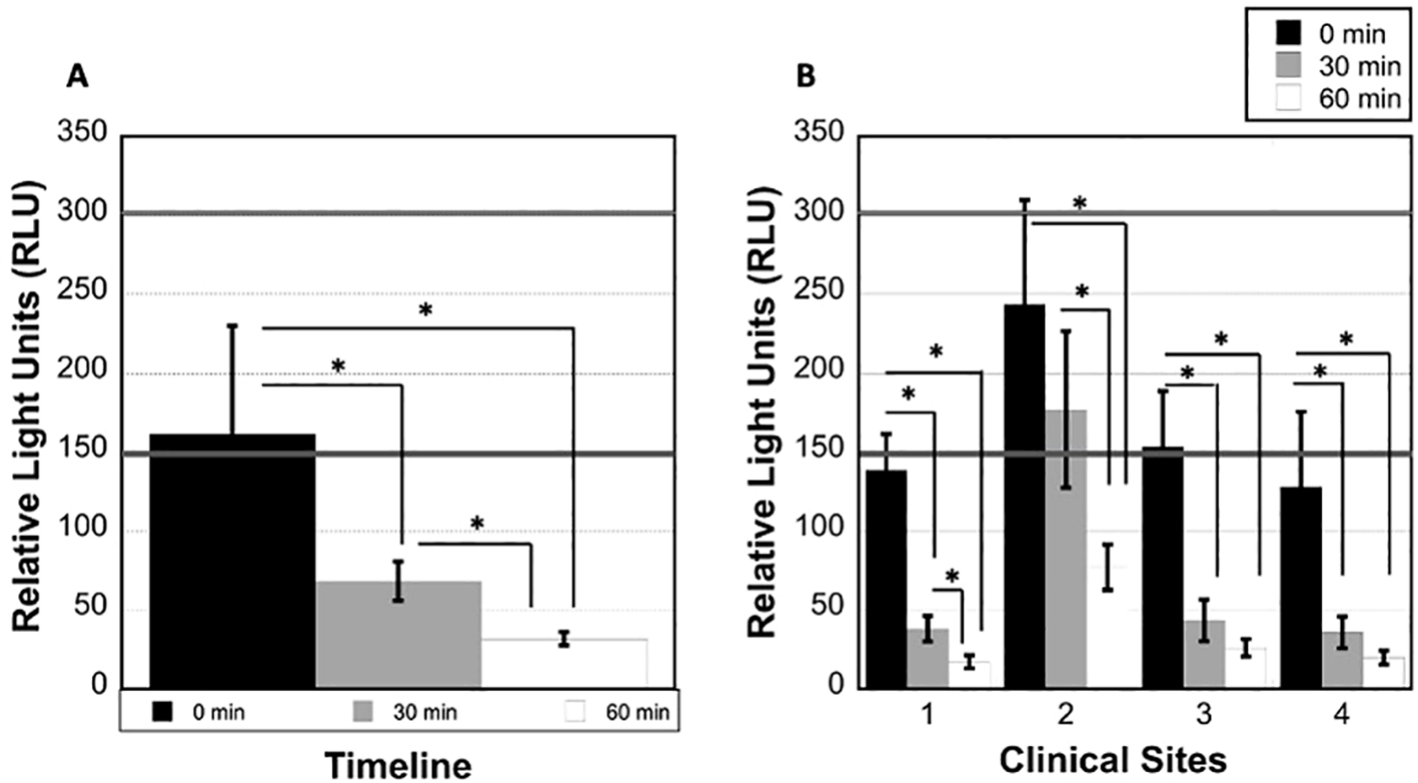
RLU levels pre- and post-treatment. The left image **(A)** shows the combined mean and variance (error bars) for RLU just prior to treatment (0 minutes) and at 30- and 60-minutes post-treatment. A statistically significant reduction in RLU was observed compared to pre-treatment levels at both times. The figure on the right **(B)** reveals similar findings for individual sites (Boyce, 2016; Shafer et al., 2019; Ejerhed et al., 2020; Jinadatha et al., 2024). Significant reductions in RLU were observed at each site, however no significant differences were observed across sites. The upper double line is the manufacturer cutoff for “dirty” ATP level and the lower solid line the cutoff for “clean.” RLU, relative light units; min, minutes. The asterisk denotes statistical significance, p < 0.05.

**Figure 2:**
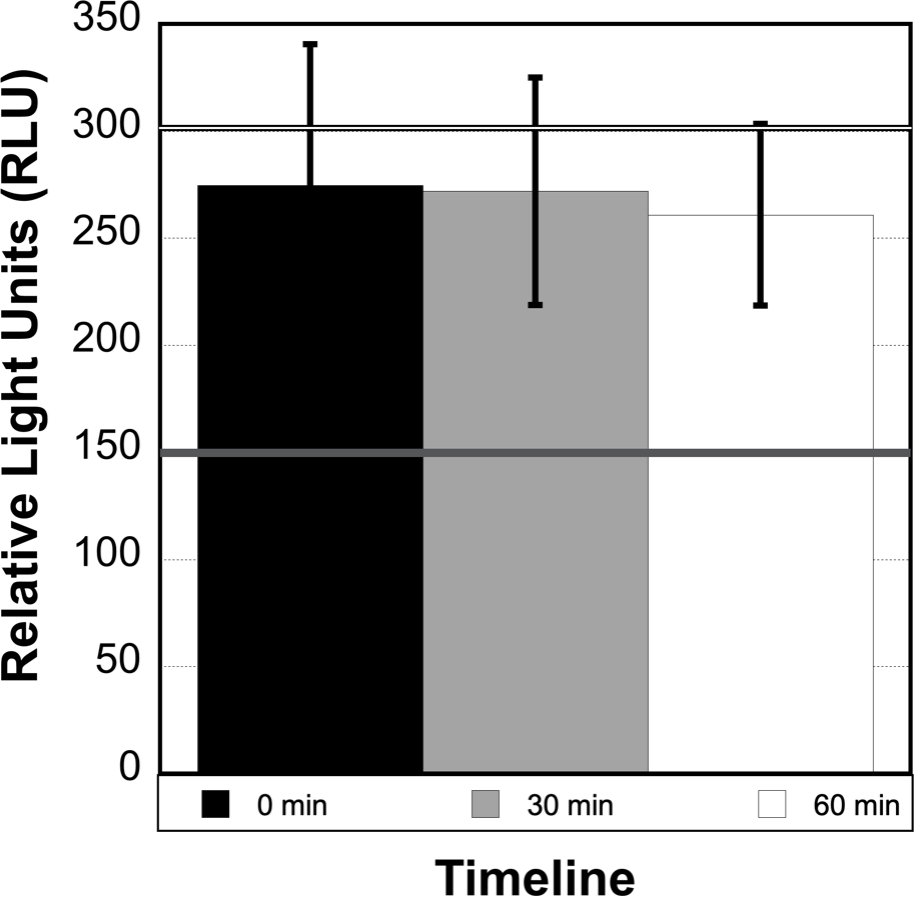
RLU levels for control samples. No significant change in RLU levels was observed for control sample at 30 or 60 minutes. The upper double line is the manufacturer cutoff for “dirty” ATP level and the lower solid line the cutoff for “clean.” RLU, relative light units; min, minutes.

**Table 1** shows mean descriptive statistics for RLU measurements pre- and post-treatment time points both overall and at individual facilities. Parametric statistics were not performed due to non-normal distribution; therefore, significance is not denoted. **Figure 1(A)** shows overall RLU findings at pre- and post-treatment periods and (B) at individual facilities; * denote statistical significance. The Wilcoxon Signed Rank tests showed a statistically significant reduction in RLU at 30 min (Z = 1461.5, p < 0.001) and 60 min post treatment (Z = 465.0, <0.001) compared to pre-treatment levels. Statistically significant reduction was also observed when comparing levels at 60 min post treatment to 30 min post treatment (Z = 7485.0, p < 0.001). Comparable findings were observed for individual sites (**Figure 1B**). Independent-samples Kruskal-Wallis tests showed no statistically significant difference in RLU levels at baseline or in the amount of shift in RLU across sites (p > 0.05), i.e. comparable reduction in RLU was observed at each facility. No statistically significant change in RLU was observed with control samples at matched time points (**Figure 2**).

**Table 1:** Pre- and Post-Dry Vapor Sterilization: RLU Measurements Across Individual Sites.

Site	Metric	Baseline (RLU)	Post 30 min (RLU)	Post 60 min (RLU)
1	Mean	138.02	38.02	16.95
	N	62	62	62
	SEM	23.27	8.10	4.07
2	Mean	243.47	176.93	76.80
	N	30	30	30
	SEM	65.91	49.62	14.29
3	Mean	153.60	43.32	25.80
	N	25	25	25
	SEM	35.03	13.15	5.51
4	Mean	127.88	36.75	19.75
	N	24	24	24
	SEM	47.61	10.04	4.39
All Sites	Mean	161.49	68.13	31.73
	N	141	141	141
	SEM	20.23	12.33	4.20

RLU, relative light units, SD, standard deviation, N, number, SEM, standard error of mean, min, minute.s

## 4 Discussion

This is one of the first experimental applications of a ultrafine aqueous “dry vapor” HOCl in a clinical setting. Significant reduction in ATP bioluminescence was demonstrated after a short treatment period. The reduction was sustained up to 60 minutes post-exposure and continued to decrease compared to 30 minutes post-exposure. All facilities showed reduction well below guidelines for terminal disinfection of hospital non-surgical areas and often to <10 RLU, consistent with surgical suite disinfection in US healthcare systems.

Manual cleaning remains an EPA regulatory requirement prior to administration of disinfection. The liability associated with current disinfection methods is compounded by factors such as antimicrobial resistance, human error in cleaning protocol execution, and exposure to toxic chemicals (Boyce, 2016). The use of HOCl in a dry vapor form broadens its potential as a powerful germicidal agent, comparable in strength to the toxic quaternary salts. Atomizing HOCl to dry vapor leads to significant reduction in bacterial growth when compared to non-aerosolized forms (Masterman, 1941).

Though the efficacy of HOCl in air purification has been demonstrated for over a century, large scale application has been limited by cumbersome machinery, solution stability issues, and application difficulty (Masterman, 1941; Overholt and Jones, 2021). Recent technological advancements enable the efficient dispersal of small droplets (< 2 microns) from a stabilized HOCl solution as novel tool for mitigating pathogens (Arcus Manufacturing, 2022; Boecker et al., 2023). HOCl has a remarkable safety profile. Recently, Muramatsu et al. (2024) demonstrated no evidence of cytotoxicity of gaseous HOCl on human nasal and bronchiolar epithelium. A study of a ten-day exposure to HOCl gas in mice found no detectable changes in blood metabolic measures nor significant lung pathology (Burd, 2020). The HOCl may remain airborne for an extended period of time, allowing for sustained disinfection of airborne pathogens; Yamuda et al. estimated the half-life of gaseous HOCL to be between 76.9 and 116 hours (Yamada et al., 2023).

This study was not without limitations. First, outcome measures were limited to ATP bioluminescence. ATP bioluminescence is poorly standardized, and each system has its own benchmark values. ATP is a proxy of organic matter and microbial contamination but is not sensitive to individual biological materials and may be influenced by high concentrations of bleach (Sanna et al., 2018). Still, ATP bioluminescence is included among CDC listed options for monitoring environmental cleaning (Guh and Carling, 2010). Further study is needed to detail specific pathogens and use of culture based quantitative assays. Second, the design was limited to an acute period, which may not translate to long-term efficacy and sustainability of the disinfection process. Further studies are needed to inform scheduling of HOCl delivery for maintenance of disinfection and to characterize airborne efficacy. The potential will be defined by more intense investigation in hospitals, nursing homes, schools, airports, biohazard situations, military installments, cruise ships— wherever there are pathogens and the use of hazardous cleaning agents are commonly utilized.

This study highlights the novel application of dry vapor HOCl in a variety of healthcare settings, demonstrating potential feasibility in real-world environments. The ease of application, which does not require licensed personnel, along with a robust safety profile and an extensive microbial kill list, reinforces the potential of HOCl as a sustainable, environmentally friendly solution in infection control.

## Data Availability

The raw data supporting the conclusions of this article will be made available by the authors, without undue reservation.
